# The relationship between low serum magnesium level and intracerebral hemorrhage hematoma expansion: Protocol for a systematic review and meta-analysis

**DOI:** 10.1097/MD.0000000000018719

**Published:** 2020-01-10

**Authors:** Rending Zhu, Xiaolu He, Yanqun Du, Nan Chen, Wei Wang, Yue Sun, Jian Sun, Wanjun Liu, Xun Wang, Chuanqin Fang

**Affiliations:** a Department of Neurology; b Department of Orthopedics; c Department of Pediatrics, The Second Affiliated Hospital of Anhui Medical University, Anhui Medical University, 678 Fu Rong Road, Hefei, Anhui Province , China.

**Keywords:** cerebral hemorrhage, hematoma expansion, magnesium, protocol, systematic review

## Abstract

**Background:**

Hematoma expansion (HE) is related to clinical deterioration and unfavorable prognosis in intracerebral hemorrhage (ICH). Some studies have revealed that low serum magnesium level is associated with larger hematoma volume at admission, HE, and unfavorable outcomes. However, the conclusions remain unsettled. The purpose of this study is to evaluate the association between low serum magnesium level and HE by meta-analysis.

**Methods:**

We will search the following electronic bibliographic databases: PubMed, Medline, Embase, Web of Science, and The Cochrane Library. Studies will be included if they reported a relationship of low serum magnesium level and HE, mortality or poor outcome.

**Results:**

The results of this study will be submitted to a peer-reviewed journal for publication.

**Conclusion:**

This will be the first systematic review and meta-analysis to evaluate the association of HE following ICH with Hypomagnesemia. We look forward to the results will offer scientific proof to predict HE for ICH patients with low serum magnesium level.

**PROSPERO registration number:**

This protocol has been registered in the PROSPERO network with number: CRD42019135995.

## 1 Introduction

Intracerebral hemorrhage (ICH) is the deadliest stroke subtype and accounts for nearly 15% of all strokes worldwide.^[1]^ It carries a poor prognosis, with a mortality up to 40% at 30 days and only 20% survivors could live independently at 6 months.^[2]^ Although Several factors have been implicated as predictors of ICH outcome including initial hematoma volume, shorter time from onset, age, and Glasgow outcome scale (GOS) score are non-modifiable at presentation.^[3–5]^ However, hematoma volume remains the strongest determinant of 30-day mortality.^[3]^ Crucially, hematoma size is variable, up to one third of ICH patients experience hematoma expansion in the first 3 to 6 hours after stroke onset,^[5,6]^ and hematoma expansion (HE) is a major determinant of early deterioration and death.^[5,7]^ It also has been regarded as an independent predictor for unfavorable outcomes after ICH.^[5,8]^ Thus, prevention of hematoma growth has become the primary goal of early ICH treatment. It is particularly important to identify and predict which patients would develop hematoma expansion. To identify an accurate and credible predictor of hematoma expansion in patients with ICH is important.^[9]^

In recent years, several studies have observed associations among admission magnesium serum level, hematoma volume, and outcome in patients with ICH. Behrouz et al^[10]^ found that hypomagnesemia is associated with severe presentation of admission but not hematoma volume and discharge outcome. Liotta et al^[11]^ indicated that lower serum magnesium level were associated with greater initial hematoma volume, the same as HE, and poor functional outcome at 3 months. Goyal et al^[12]^ analysis showed that higher admission serum magnesium level were strongly correlated to smaller initial hematoma volume, as well as admission ICH score and favorable functional outcome in patients with ICH. Finally, Han et al^[13]^ results show that higher baseline serum magnesium level was significantly associated with decreased mortality at 3 months in ICH patients, but they did not found the same association between serum magnesium and poor functional outcome at 3months. So far, the role of serum magnesium in HE and outcome following ICH remains limited and controversial. Thus, we carry out this study to assess the predictive ability of low serum magnesium level for HE or poor outcome after ICH. To our knowledge, this is the first meta-analysis to assess the correlations of low serum magnesium level with HE and outcome in ICH patients.

## 2 Methods

### 2.1 Standards

This protocol will be performed comply with the Preferred Reporting Items for Systematic Reviews and Meta-Analyses Protocols (PRISMA-P) guidelines.^[14]^

### 2.2 Ethical issues

Ethical approval is not required as this study based on aggregate data and will be not involve humans.

### 2.3 Registration

The protocol has been registered on PROSPERO with number: CRD42019135995.

### 2.4 Data sources and search strategy

We will systematically search the following databases: PubMed, Medline, EMBASE, Web of Science, and The Cochrane Library. The language was restricted to English.

The reference lists and related articles in each identified publication were reviewed to identify other potential studies.

The search for PubMed will be performed using multiple combinations of the following terms:

1. serum magnesium OR blood magnesium OR magnesium OR hypomagnesemia; AND
2. hematoma volume OR extent of bleeding OR hematoma expansion OR hematoma enlargement OR hematoma growth; AND
3. intracerebral hemorrhage OR cerebral hemorrhage AND
4. association OR relationship. Search term will be adapted to other databases based on their specific requirements for each database.

### 2.5 Inclusion and exclusion criteria

Studies will be included if they meet the standard as follows

1. retrospective studies, randomized control trials, case-control, and cohort;
2. Intracerebral hemorrhage diagnosed by CT;
3. data can be used for quantitative analysis. Secondary intracerebral hemorrhage, studies that did not report serum magnesium data, hematoma expansion, and clinical outcomes will be excluded.

### 2.6 Data extraction and quality assessment

**Figure 1:**
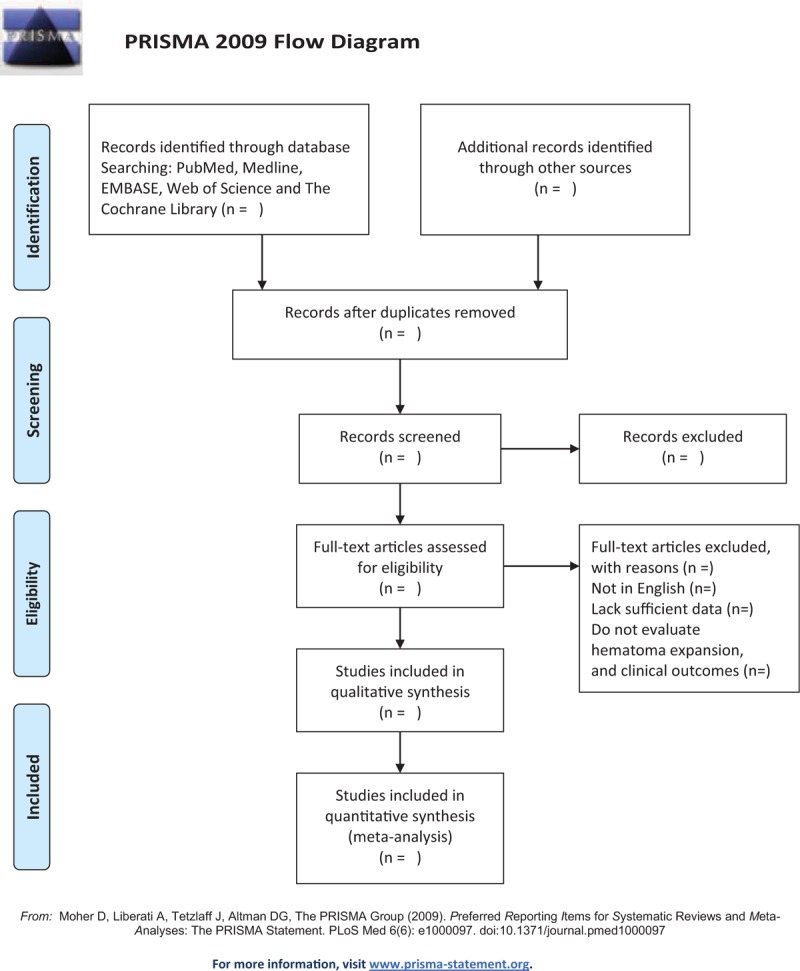
Flow chart of literature screening process. From: Moher D, Liberati A, Tetzlaff J, Altman DG, The PRISMA Group (2009). Preferred Reporting Items for Systematic Reviews and Meta-Analyses: The PRISMA Statement. PLoS Med 6(6): e1000097. doi:10.1371/journal.pmed1000097.

Characteristics and data will be extracted by using a standardized form after 2 researchers independently reviewed the included studies. The procedure of selection will be summarized base on PRISMA flow diagram (Fig. 1). The collected data were as follows: author, publication year, country, design, study period, effective sample size, onset to CT scan time, definition of HE, follow up CT time, demographic information, and outcome measurements.

Two researchers independently assessed methodological quality using the Newcastle–Ottawa Scale. Discrepancies will be solved by discussion and consensus.

Definition hematoma expansion defined as relative hemorrhage growth >33% or absolute hemorrhage growth > 6 ml from initial CT scan to follow-up CT scan.^[15]^ Death and poor clinical outcome as outcome indicators measured by the Glasgow Outcome Scale (GOS) score, or modified Rankin score (mRS).

### 2.7 Statistical analysis

Review Manager software (version 5.3.3 Cochrane Collaboration) will be used for all statistical analyses in this meta-analysis. We will use odds ratio (OR) and its 95% confidence intervals (CIs) selected as the effect size to quantify the strength of association between low serum magnesium level and HE, death, or poor clinical outcome. Chi-Squared and Cochran-*Q* test will be used to assess the heterogeneity across included studies, substantial heterogeneity will be determined if *I*^2^ > 50%. Summary receiver operator characteristics (SROC) curve was conducted to evaluate the accuracy of low serum magnesium level for predicting hematoma expansion. The publication bias across included studies will be evaluated by using a funnel plot asymmetry test, and significant publication bias was identified if *P* < .05.

## 3 Discussion

Intracerebral hemorrhage (ICH) accounts for 10% to 15% of all strokes and is associated with 40% early mortality and 80% disability.^[2,16,17]^ Early neurological deterioration, poor outcomes of ICH are associated with large initial ICH volumes and hematoma expansion (HE)^[15,18,19]^ Recently, more and more studies have investigated the roles for magnesium in preventing hematoma growth and improving clinical outcome in ICH. However, the results of these studies are inconsistent and controversial. Hence, we hope this review will determine whether serum magnesium can be used as a biomarker for hematoma expansion and poor outcome. However, more prospective and larger sample size studies are needed to verify our findings in the future due to the possible heterogeneity among studies and limitations of studies in our analysis.

## Author contributions

**Conceptualization:** Rending Zhu, Xiaolu He, Chuanqin Fang.

**Data curation:** Rending Zhu, Xiaolu He, Yanqun Du.

**Formal analysis:** Rending Zhu, Yue Sun, Wanjun Liu.

**Funding acquisition:** Xun Wang, Chuanqin Fang.

**Methodology:** Rending Zhu, Nan Chen, Wei Wang, Jian Sun.

**Supervision:** Chuanqin Fang.

**Writing – original draft:** Rending Zhu.

**Writing – review & editing:** Rending Zhu, Xiaolu He, Xun Wang, Chuanqin Fang.

## References

[1] Qureshi AI Mendelow AD Hanley DF Intracerebral haemorrhage. Lancet 2009;373:1632–44. 19427958 10.1016/S0140-6736(09)60371-8 PMC3138486

[2] van Asch CJ Luitse MJ Rinkel GJ Incidence, case fatality, and functional outcome of intracerebral haemorrhage over time, according to age, sex, and ethnic origin: a systematic review and meta-analysis. Lancet Neurol 2010;9:167–76. 20056489 10.1016/S1474-4422(09)70340-0

[3] Broderick JP Brott TG Duldner JE Volume of intracerebral hemorrhage. A powerful and easy-to-use predictor of 30-day mortality. Stroke 1993;24:987–93. 8322400 10.1161/01.str.24.7.987

[4] Hemphill JC Bonovich DC Besmertis L The ICH score: a simple, reliable grading scale for intracerebral hemorrhage. Stroke 2001;32:891–7. 11283388 10.1161/01.str.32.4.891

[5] Davis SM Broderick J Hennerici M Hematoma growth is a determinant of mortality and poor outcome after intracerebral hemorrhage. Neurology 2006;66:1175–81. 16636233 10.1212/01.wnl.0000208408.98482.99

[6] Steiner T Bösel J Options to restrict hematoma expansion after spontaneous intracerebral hemorrhage. Stroke 2010;41:402–9. 20044536 10.1161/STROKEAHA.109.552919

[7] Kazui S Naritomi H Yamamoto H Enlargement of spontaneous intracerebral hemorrhage. Incidence and time course. Stroke 1996;27:1783–7. 8841330 10.1161/01.str.27.10.1783

[8] Delcourt C Huang Y Arima H Hematoma growth and outcomes in intracerebral hemorrhage: the INTERACT1 study. Neurology 2012;79:314–9. 22744655 10.1212/WNL.0b013e318260cbba

[9] Becker K Tirschwell D Intraparenchymal hemorrhage, bleeding, hemostasis, and the utility of CT angiography. Int J Stroke 2008;3:11–3. 18705909 10.1111/j.1747-4949.2008.00179.x

[10] Behrouz R Hafeez S Mutgi SA Hypomagnesemia in Intracerebral Hemorrhage. World Neurosurg 2015;84:1929–32. 26341430 10.1016/j.wneu.2015.08.036

[11] Liotta E Prabhakaran S Sangha R Magnesium, hemostasis, and outcomes in patients with intracerebral hemorrhage. Neurology 2017;89:813–9. 28747450 10.1212/WNL.0000000000004249 PMC5580864

[12] Goyal N Tsivgoulis G Malhotra K Serum Magnesium levels and outcomes in patients with acute spontaneous intracerebral hemorrhage. J Am Heart Assoc 2018;7 (8.):10.1161/JAHA.118.008698 PMC6015418 29654197

[13] Han X You S Huang Z Prognostic significance of serum magnesium in acute intracerebral hemorrhage patients. Curr Neurovasc Res 2019;16:123–8. 30977445 10.2174/1567202616666190412124539

[14] Shamseer L Moher D Clarke M Preferred reporting items for systematic review and meta-analysis protocols (PRISMA-P) 2015: elaboration and explanation. BMJ (Clinical research ed) 2015;350:g7647. 10.1136/bmj.g7647 25555855

[15] Dowlatshahi D Demchuk AM Flaherty ML Defining hematoma expansion in intracerebral hemorrhage: relationship with patient outcomes. Neurology 2011;76:1238–44. 21346218 10.1212/WNL.0b013e3182143317 PMC3068004

[16] Sacco S Marini C Toni D Incidence and 10-year survival of intracerebral hemorrhage in a population-based registry. Stroke 2009;40:394–9. 19038914 10.1161/STROKEAHA.108.523209

[17] Rost NS Smith EE Chang Y Prediction of functional outcome in patients with primary intracerebral hemorrhage: the FUNC score. Stroke 2008;39:2304–9. 18556582 10.1161/STROKEAHA.107.512202

[18] Al-Mufti F Amuluru K Changa A Traumatic brain injury and intracranial hemorrhage-induced cerebral vasospasm: a systematic review. Neurosurg Focus 2017;43:E14. 10.3171/2017.8.FOCUS17431 29088959

[19] Dowlatshahi D Wasserman JK Momoli F Evolution of computed tomography angiography spot sign is consistent with a site of active hemorrhage in acute intracerebral hemorrhage. Stroke 2014;45:277–80. 24178918 10.1161/STROKEAHA.113.003387

